# The Lancet Psychiatry Commission on Transforming Mental Health Implementation Research

**DOI:** 10.1192/j.eurpsy.2025.43

**Published:** 2025-08-26

**Authors:** J. Marsh

**Affiliations:** The Lancet Psychiatry, London, United Kingdom

## Abstract

**Abstract:**

Effective approaches exist to prevent and treat mental illness and to promote mental health but most people who could benefit from evidence-based interventions (policies, programmes, and individual-level practices or services) do not receive them. Too often, research produces interventions and implementation strategies that are difficult to scale owing to misalignment with the political, cultural, policy, system, community, provider, and individual realities of real-world settings. The *Lancet Psychiatry* Commission on Transforming Mental Health Implementation Research considers strategies for changing how research is done to produce more actionable evidence. It examines how to integrate research and real-world implementation; centre equity in mental health intervention and implementation research; apply a complexity science lens to mental health research; expand designs beyond the randomised clinical trial; and value transdisciplinarity across endeavours. Most mental health implementation research has been done in high-income countries but the Commission’s recommendations incorporate research from low-income and middle-income countries and call for strategies to expand mental health implementation research globally.

**Disclosure of Interest:**

J. Marsh Employee of: Elsevier

